# Ethyl 3-(4-hydroxy­phen­oxy)-2-(4-methoxy­phen­yl)acrylate

**DOI:** 10.1107/S160053680803571X

**Published:** 2008-11-08

**Authors:** Jin Hou

**Affiliations:** a Department of Chemistry and Chemical Engineering, South-East University, Nanjing 211189, and Nantong Entry-Exit Inspection and Quarantine Bureau, Nantong 226005, People’s Republic of China

## Abstract

In the title compound, C_18_H_18_O_5_, the dihedral angle between the two benzene rings is 55.2 (3)°. The ethyl acrylate linkage is planar and forms dihedral angles of 21.3 (3) and 41.0 (3)°, respectively, with the hydroxy­phenyl and methoxy­phenyl rings. In the crystal structure, mol­ecules are linked into zigzag chains along the *b* axis by O—H⋯O hydrogen bonds.

## Related literature

For general background, see: Huang *et al.* (2008 ▶); Li *et al.* (2008 ▶); Liu *et al.* (2008 ▶); Shi *et al.* (2008 ▶); Xiao *et al.* (2008 ▶). For bond-length data, see: Allen *et al.* (1987 ▶).
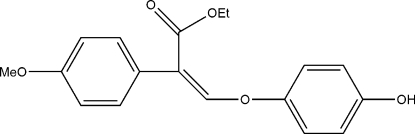


## Experimental

### 

#### Crystal data

C_18_H_18_O_5_
*M* *_r_* = 314.33
Orthorhombic, 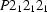

*a* = 7.4773 (16) Å
*b* = 11.661 (2) Å
*c* = 18.417 (4) Å
*V* = 1605.8 (6) Å^3^
*Z* = 4
Mo *K*α radiation
μ = 0.10 mm^−1^
*T* = 298 (2) K
0.40 × 0.30 × 0.20 mm

#### Data collection

Bruker SMART CCD area-detector diffractometer
Absorption correction: multi-scan (*SADABS*; Bruker, 2001 ▶) *T*_min_ = 0.963, *T*_max_ = 0.981
5527 measured reflections
1822 independent reflections
1486 reflections with *I* > 2σ(*I*)
*R*_int_ = 0.032

#### Refinement

*R*[*F* ^2^ > 2σ(*F* ^2^)] = 0.051
*wR*(*F* ^2^) = 0.134
*S* = 1.05
1822 reflections
211 parameters
H-atom parameters constrained
Δρ_max_ = 0.26 e Å^−3^
Δρ_min_ = −0.34 e Å^−3^

### 

Data collection: *SMART* (Bruker, 2007 ▶); cell refinement: *SAINT* (Bruker, 2007 ▶); data reduction: *SAINT*; program(s) used to solve structure: *SHELXTL* (Sheldrick, 2008 ▶); program(s) used to refine structure: *SHELXTL*; molecular graphics: *SHELXTL*; software used to prepare material for publication: *SHELXTL*.

## Supplementary Material

Crystal structure: contains datablocks global, I. DOI: 10.1107/S160053680803571X/ci2693sup1.cif

Structure factors: contains datablocks I. DOI: 10.1107/S160053680803571X/ci2693Isup2.hkl

Additional supplementary materials:  crystallographic information; 3D view; checkCIF report

## Figures and Tables

**Table 1:** Hydrogen-bond geometry (Å, °)

*D*—H⋯*A*	*D*—H	H⋯*A*	*D*⋯*A*	*D*—H⋯*A*
O4—H4⋯O1^i^	0.82	2.00	2.812 (3)	169

Symmetry code: (i) 
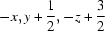
.

**Fig. 1.**
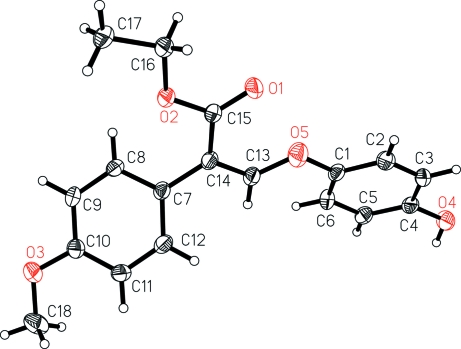
The molecular structure of the title compound, showing 30% probability displacement ellipsoids and the atom-numbering scheme.

**Fig. 2.**
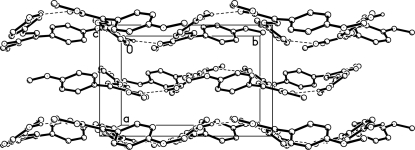
The crystal structure of the title compound, viewed along the c axis. Hydrogen bonds are shown as dashed lines.

## supplementary crystallographic information

### Comment

Phenylacetate and styrene derivetives are important for their extensive biological activities. Recently a great deal of such kinds of compounds were synthesized, which were found to exhibit good activities (Huang *et al.*, 2008; Li *et al.*, 2008; Liu *et al.*, 2008; Shi *et al.*, 2008; Xiao *et al.*, 2008)

Bond lengths in the title compound (Fig.1) are within normal ranges (Allen *et al.*, 1987). The dihedral angle between the C1—C6 and C7—C12 benzene rings is 55.2 (3)°. The O1/O2/C13—C17 plane forms dihedral angles of 21.3 (3)° and 41.0 (3)°, respectively, with C1—C6 and C7—C12 benzene rings. In the crytal structure, O—H···O hydrogen bonds (Table 1) link the molecules into zigzag chains along the *b* axis (Fig. 2).

### Experimental

Equimolar ethyl 3-bromo-2-(4-methoxyphenyl)acrylate and hydroquinone reacted in chloroform overnight, gave the title compound in high yield (88%). Colourless crystals of the title compound were grown by slow evaparation of a methanol solution.

### Refinement

H atoms were positioned geometrically (O—H = 0.82 Å and C—H = 0.93 Å) and refined using a riding model, with *U*_iso_(H) = 1.2*U*_eq_(C) and 1.5*U*_eq_(O). In the absence of significant anomalous scattering, Friedel pairs were merged prior to the final refinement.

### Crystal data

**Table:**

C_18_H_18_O_5_	*F*_000_ = 664
*M**_r_* = 314.33	*D*_x_ = 1.304 Mg m^−^^3^
Orthorhombic, *P*2_1_2_1_2_1_	Mo *K*α radiation λ = 0.71073 Å
Hall symbol: P 2ac 2ab	Cell parameters from 1213 reflections
*a* = 7.4773 (16) Å	θ = 3.2–26.1º
*b* = 11.661 (2) Å	µ = 0.10 mm^−^^1^
*c* = 18.417 (4) Å	*T* = 298 (2) K
*V* = 1605.8 (6) Å^3^	Prism, colourless
*Z* = 4	0.40 × 0.30 × 0.20 mm

### Data collection

**Table:**

Bruker SMART CCD area-detector diffractometer	1822 independent reflections
Radiation source: fine-focus sealed tube	1486 reflections with *I* > 2σ(*I*)
Monochromator: graphite	*R*_int_ = 0.032
*T* = 298(2) K	θ_max_ = 26.0º
φ and ω scans	θ_min_ = 2.1º
Absorption correction: multi-scan(SADABS; Bruker, 2001)	*h* = −9→9
*T*_min_ = 0.963, *T*_max_ = 0.981	*k* = −10→14
5527 measured reflections	*l* = −22→22

### Refinement

**Table:**

Refinement on *F*^2^	Secondary atom site location: difference Fourier map
Least-squares matrix: full	Hydrogen site location: inferred from neighbouring sites
*R*[*F*^2^ > 2σ(*F*^2^)] = 0.051	H-atom parameters constrained
*wR*(*F*^2^) = 0.134	*w* = 1/[σ^2^(*F*_o_^2^) + (0.0689*P*)^2^ + 0.4887*P*] where *P* = (*F*_o_^2^ + 2*F*_c_^2^)/3
*S* = 1.05	(Δ/σ)_max_ = 0.001
1822 reflections	Δρ_max_ = 0.26 e Å^−^^3^
211 parameters	Δρ_min_ = −0.34 e Å^−^^3^
Primary atom site location: structure-invariant direct methods	Extinction correction: none

### Special details

**Table:**

Geometry. All e.s.d.'s (except the e.s.d. in the dihedral angle between two l.s. planes) are estimated using the full covariance matrix. The cell e.s.d.'s are taken into account individually in the estimation of e.s.d.'s in distances, angles and torsion angles; correlations between e.s.d.'s in cell parameters are only used when they are defined by crystal symmetry. An approximate (isotropic) treatment of cell e.s.d.'s is used for estimating e.s.d.'s involving l.s. planes.
Refinement. Refinement of *F*^2^ against ALL reflections. The weighted *R*-factor *wR* and goodness of fit *S* are based on *F*^2^, conventional *R*-factors *R* are based on *F*, with *F* set to zero for negative *F*^2^. The threshold expression of *F*^2^ > σ(*F*^2^) is used only for calculating *R*-factors(gt) *etc*. and is not relevant to the choice of reflections for refinement. *R*-factors based on *F*^2^ are statistically about twice as large as those based on *F*, and *R*- factors based on ALL data will be even larger.

### Fractional atomic coordinates and isotropic or equivalent isotropic displacement parameters (Å^2^)

**Table:**

	*x*	*y*	*z*	*U*_iso_*/*U*_eq_	
C1	0.0040 (5)	0.4964 (3)	0.73124 (16)	0.0401 (8)	
C2	0.0773 (6)	0.4306 (3)	0.78656 (17)	0.0485 (9)	
H2	0.1463	0.3669	0.7750	0.058*	
C3	0.0490 (6)	0.4589 (3)	0.85909 (18)	0.0489 (10)	
H3	0.0990	0.4144	0.8957	0.059*	
C4	−0.0543 (6)	0.5537 (3)	0.87635 (17)	0.0444 (9)	
C5	−0.1311 (5)	0.6181 (3)	0.82193 (17)	0.0454 (9)	
H5	−0.2019	0.6810	0.8335	0.054*	
C6	−0.1024 (5)	0.5887 (3)	0.74921 (17)	0.0471 (9)	
H6	−0.1556	0.6319	0.7126	0.057*	
C7	0.0419 (5)	0.6087 (3)	0.47594 (16)	0.0370 (7)	
C8	−0.0503 (5)	0.5959 (3)	0.41071 (17)	0.0407 (8)	
H8	−0.0970	0.5246	0.3984	0.049*	
C9	−0.0734 (5)	0.6872 (3)	0.36402 (17)	0.0462 (9)	
H9	−0.1336	0.6762	0.3204	0.055*	
C10	−0.0083 (5)	0.7946 (3)	0.38124 (18)	0.0441 (9)	
C11	0.0842 (5)	0.8107 (3)	0.44550 (19)	0.0463 (9)	
H11	0.1287	0.8826	0.4578	0.056*	
C12	0.1095 (5)	0.7176 (3)	0.49137 (19)	0.0440 (9)	
H12	0.1741	0.7282	0.5340	0.053*	
C13	0.0301 (5)	0.5385 (3)	0.60130 (17)	0.0415 (8)	
H13	0.0000	0.6138	0.6123	0.050*	
C14	0.0590 (5)	0.5141 (3)	0.52943 (17)	0.0394 (8)	
C15	0.1049 (5)	0.3973 (3)	0.50927 (17)	0.0397 (8)	
C16	0.1644 (7)	0.2682 (3)	0.41167 (18)	0.0513 (10)	
H16A	0.0775	0.2121	0.4281	0.062*	
H16B	0.2814	0.2462	0.4297	0.062*	
C17	0.1658 (6)	0.2743 (3)	0.33027 (17)	0.0549 (10)	
H17A	0.0455	0.2826	0.3128	0.082*	
H17B	0.2170	0.2053	0.3109	0.082*	
H17C	0.2357	0.3390	0.3150	0.082*	
C18	−0.0024 (7)	0.9941 (3)	0.3518 (3)	0.0668 (13)	
H18A	−0.0656	1.0139	0.3954	0.100*	
H18B	−0.0374	1.0450	0.3134	0.100*	
H18C	0.1239	1.0008	0.3600	0.100*	
O5	0.0407 (4)	0.4650 (3)	0.65807 (14)	0.0662 (8)	
O1	0.1315 (4)	0.3188 (2)	0.55214 (12)	0.0541 (7)	
O2	0.1174 (4)	0.3810 (2)	0.43764 (12)	0.0468 (7)	
O3	−0.0441 (5)	0.8795 (2)	0.33197 (14)	0.0618 (8)	
O4	−0.0756 (5)	0.5803 (2)	0.94804 (12)	0.0601 (8)	
H4	−0.1019	0.6483	0.9520	0.090*	

### Atomic displacement parameters (Å^2^)

**Table:**

	*U*^11^	*U*^22^	*U*^33^	*U*^12^	*U*^13^	*U*^23^
C1	0.047 (2)	0.0446 (17)	0.0288 (16)	−0.0058 (17)	−0.0002 (15)	−0.0054 (14)
C2	0.062 (2)	0.0465 (19)	0.0373 (17)	−0.003 (2)	0.0026 (18)	−0.0020 (15)
C3	0.071 (3)	0.0439 (18)	0.0320 (16)	−0.008 (2)	−0.0065 (18)	0.0043 (15)
C4	0.060 (2)	0.0450 (19)	0.0281 (15)	−0.011 (2)	0.0040 (16)	−0.0038 (14)
C5	0.053 (2)	0.0458 (19)	0.0379 (18)	0.0008 (19)	0.0010 (17)	−0.0091 (16)
C6	0.054 (2)	0.056 (2)	0.0313 (16)	0.002 (2)	−0.0039 (16)	0.0043 (16)
C7	0.0403 (17)	0.0406 (17)	0.0302 (15)	−0.0005 (17)	0.0009 (15)	−0.0029 (14)
C8	0.046 (2)	0.0405 (18)	0.0352 (17)	−0.0062 (17)	−0.0021 (16)	−0.0060 (14)
C9	0.058 (2)	0.052 (2)	0.0282 (15)	−0.004 (2)	−0.0067 (16)	−0.0010 (15)
C10	0.048 (2)	0.0461 (19)	0.0376 (18)	−0.0014 (18)	0.0041 (17)	−0.0001 (15)
C11	0.053 (2)	0.0395 (17)	0.0464 (19)	−0.0077 (18)	0.0007 (18)	−0.0043 (16)
C12	0.045 (2)	0.049 (2)	0.0385 (18)	−0.0055 (18)	−0.0048 (17)	−0.0065 (16)
C13	0.048 (2)	0.0437 (18)	0.0329 (16)	0.0006 (18)	−0.0030 (15)	−0.0030 (15)
C14	0.0419 (19)	0.0441 (18)	0.0321 (16)	−0.0035 (17)	−0.0041 (16)	−0.0026 (14)
C15	0.048 (2)	0.0404 (17)	0.0303 (16)	−0.0023 (18)	0.0012 (16)	−0.0017 (14)
C16	0.081 (3)	0.0400 (19)	0.0332 (17)	0.001 (2)	0.002 (2)	−0.0028 (15)
C17	0.075 (3)	0.055 (2)	0.0348 (18)	−0.005 (2)	0.0052 (19)	−0.0094 (17)
C18	0.068 (3)	0.047 (2)	0.086 (3)	0.000 (2)	0.000 (3)	0.014 (2)
O5	0.080 (2)	0.0751 (18)	0.0436 (14)	0.0007 (19)	0.0009 (15)	−0.0010 (14)
O1	0.087 (2)	0.0428 (13)	0.0328 (12)	0.0010 (15)	0.0023 (14)	0.0029 (11)
O2	0.0716 (17)	0.0392 (12)	0.0296 (11)	0.0028 (13)	0.0012 (13)	−0.0037 (10)
O3	0.092 (2)	0.0451 (14)	0.0488 (14)	−0.0026 (16)	−0.0042 (16)	0.0095 (12)
O4	0.098 (2)	0.0527 (15)	0.0292 (12)	−0.0039 (18)	0.0073 (14)	−0.0048 (11)

### Geometric parameters (Å, °)

**Table:**

C1—C6	1.379 (5)	C11—C12	1.388 (5)
C1—C2	1.388 (5)	C11—H11	0.93
C1—O5	1.423 (4)	C12—H12	0.93
C2—C3	1.392 (5)	C13—O5	1.354 (4)
C2—H2	0.93	C13—C14	1.371 (4)
C3—C4	1.386 (5)	C13—H13	0.93
C3—H3	0.93	C14—C15	1.454 (4)
C4—O4	1.366 (4)	C15—O1	1.225 (4)
C4—C5	1.377 (5)	C15—O2	1.336 (4)
C5—C6	1.399 (5)	C16—O2	1.444 (4)
C5—H5	0.93	C16—C17	1.501 (5)
C6—H6	0.93	C16—H16A	0.97
C7—C8	1.393 (5)	C16—H16B	0.97
C7—C12	1.396 (4)	C17—H17A	0.96
C7—C14	1.484 (4)	C17—H17B	0.96
C8—C9	1.379 (5)	C17—H17C	0.96
C8—H8	0.93	C18—O3	1.420 (5)
C9—C10	1.381 (5)	C18—H18A	0.96
C9—H9	0.93	C18—H18B	0.96
C10—O3	1.369 (4)	C18—H18C	0.96
C10—C11	1.384 (5)	O4—H4	0.82
			
C6—C1—C2	118.9 (3)	C11—C12—H12	118.7
C6—C1—O5	122.7 (3)	C7—C12—H12	118.7
C2—C1—O5	118.5 (3)	O5—C13—C14	127.3 (3)
C1—C2—C3	120.9 (4)	O5—C13—H13	116.4
C1—C2—H2	119.6	C14—C13—H13	116.4
C3—C2—H2	119.6	C13—C14—C15	118.5 (3)
C4—C3—C2	119.6 (3)	C13—C14—C7	118.3 (3)
C4—C3—H3	120.2	C15—C14—C7	123.2 (3)
C2—C3—H3	120.2	O1—C15—O2	121.3 (3)
O4—C4—C5	122.1 (3)	O1—C15—C14	125.0 (3)
O4—C4—C3	117.9 (3)	O2—C15—C14	113.7 (3)
C5—C4—C3	120.0 (3)	O2—C16—C17	106.8 (3)
C4—C5—C6	120.0 (3)	O2—C16—H16A	110.4
C4—C5—H5	120.0	C17—C16—H16A	110.4
C6—C5—H5	120.0	O2—C16—H16B	110.4
C1—C6—C5	120.6 (3)	C17—C16—H16B	110.4
C1—C6—H6	119.7	H16A—C16—H16B	108.6
C5—C6—H6	119.7	C16—C17—H17A	109.5
C8—C7—C12	116.9 (3)	C16—C17—H17B	109.5
C8—C7—C14	122.3 (3)	H17A—C17—H17B	109.5
C12—C7—C14	120.7 (3)	C16—C17—H17C	109.5
C9—C8—C7	121.1 (3)	H17A—C17—H17C	109.5
C9—C8—H8	119.4	H17B—C17—H17C	109.5
C7—C8—H8	119.4	O3—C18—H18A	109.5
C8—C9—C10	120.9 (3)	O3—C18—H18B	109.5
C8—C9—H9	119.6	H18A—C18—H18B	109.5
C10—C9—H9	119.6	O3—C18—H18C	109.5
O3—C10—C9	115.8 (3)	H18A—C18—H18C	109.5
O3—C10—C11	124.5 (3)	H18B—C18—H18C	109.5
C9—C10—C11	119.7 (3)	C13—O5—C1	123.8 (3)
C10—C11—C12	118.9 (3)	C15—O2—C16	118.3 (3)
C10—C11—H11	120.6	C10—O3—C18	117.8 (3)
C12—C11—H11	120.6	C4—O4—H4	109.5
C11—C12—C7	122.5 (3)		
			
C6—C1—C2—C3	−1.9 (6)	C14—C7—C12—C11	−174.8 (3)
O5—C1—C2—C3	178.6 (3)	O5—C13—C14—C15	0.5 (6)
C1—C2—C3—C4	0.2 (6)	O5—C13—C14—C7	−179.4 (3)
C2—C3—C4—O4	−178.4 (4)	C8—C7—C14—C13	−134.7 (4)
C2—C3—C4—C5	1.2 (6)	C12—C7—C14—C13	41.3 (5)
O4—C4—C5—C6	178.7 (4)	C8—C7—C14—C15	45.3 (5)
C3—C4—C5—C6	−1.0 (6)	C12—C7—C14—C15	−138.6 (4)
C2—C1—C6—C5	2.2 (6)	C13—C14—C15—O1	−4.9 (6)
O5—C1—C6—C5	−178.4 (3)	C7—C14—C15—O1	175.0 (4)
C4—C5—C6—C1	−0.7 (6)	C13—C14—C15—O2	175.4 (3)
C12—C7—C8—C9	−0.2 (5)	C7—C14—C15—O2	−4.7 (5)
C14—C7—C8—C9	176.0 (3)	C14—C13—O5—C1	−178.3 (4)
C7—C8—C9—C10	−1.1 (6)	C6—C1—O5—C13	23.2 (6)
C8—C9—C10—O3	−178.0 (4)	C2—C1—O5—C13	−157.3 (4)
C8—C9—C10—C11	1.2 (6)	O1—C15—O2—C16	−0.4 (6)
O3—C10—C11—C12	179.1 (4)	C14—C15—O2—C16	179.3 (3)
C9—C10—C11—C12	0.0 (6)	C17—C16—O2—C15	179.1 (3)
C10—C11—C12—C7	−1.4 (6)	C9—C10—O3—C18	171.3 (4)
C8—C7—C12—C11	1.5 (6)	C11—C10—O3—C18	−7.8 (6)

### Hydrogen-bond geometry (Å, °)

**Table:**

*D*—H···*A*	*D*—H	H···*A*	*D*···*A*	*D*—H···*A*
O4—H4···O1^i^	0.82	2.00	2.812 (3)	169

Symmetry codes: (i) −*x*, *y*+1/2, −*z*+3/2.
